# Anti-inflammatory new coumarin from the *Ammi majus L*

**DOI:** 10.1186/2191-2858-2-1

**Published:** 2012-01-12

**Authors:** Yasser Abdelaal Selim, Nabil Hassan Ouf

**Affiliations:** 1 Faculty of Specific Education, Zagazig University, Zagazig, Egypt; 2 Chemistry Department, Faculty of Science, Zagazig University, Zagazig, Egypt

**Keywords:** phytochemistry, *Ammi majus L*., anti-viral activity, natural products, anti-inflammatory activity, steroids

## Abstract

Investigation of the aerial parts of the Egyptian medicinal plant *Ammi majus L*. led to isolation of new coumarin, 6-hydroxy-7-methoxy-4 methyl coumarin **(2) **and 6-hydroxy-7-methoxy coumarin **(3)**; this is the first time they have been isolated from this plant. The structures of the compounds (**2 **&**3**) were elucidated by spectroscopic data interpretation and showed anti-inflammatory and anti-viral activity.

**Graphical abstract:**

An efficient, one-new coumarin (**2**) was isolated from the aerial parts of the *A. Majus L*. was evaluated for their anti-viral and anti-inflammatory activities.

## 1. Introduction

Fructus Ammi Majoris consists of the dried ripe fruits of *Ammi majus L*. (Apiaceae) [1,2]; originating Egypt, and widely distributed in Europe, the Mediterranean region, and western Asia, now cultivated in India [2]. This is widely used for the treatment of skin disorders such as psoriasis and vitiligo (acquired leukoderma) [1,3-6], and of vitiligo [1]. It is used as an emmenagogue to regulate menstruation, as a diuretic, and for treatment of leprosy, kidney stones, and urinary tract infections [7]. Numerous clinical trials have assessed the efficacy of Fructus Ammi Majoris andxanthotoxin for the treatment of vitiligo, psoriasis, and hypopigmentation tinea versicolor [4-6,8-11].

## 2. Results and discussion

### 2.1. Chemistry

**Figure 1:**
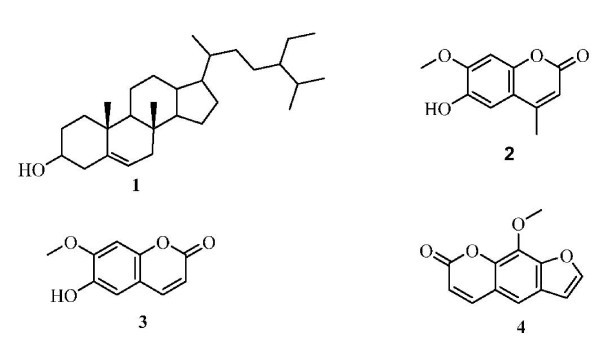
The structure of isolated compounds.

The use of natural products in drug manufacturing is an ancient and well-established practice [12]. Egyptian medicinal plants are well known by their diverse uses in traditional folk medicine to cure various ailments including infectious diseases and known producers of pharmacological and anti-viral agents [13]*A. majus L*. is contraindicated in diseases associated with photosensitivity, cataract, invasive squamous-cell cancer, known sensitivity to xanthotoxin (psoralens), and in children under the age of 12 years [14]. The fruits are also contraindicated in pregnancy, nursing, tuberculosis, liver and kidney diseases, human immunodeficiency virus (HIV) infections and other autoimmune diseases [15]. In this study, the isolated compounds (**2-4**) from *A. majus L*. were evaluated for their anti-viral activity (Figure 1). The major constituents are furanocoumarins, the principal compound beingxanthotoxin (methoxsalen, 8-methoxypsorale [8-MOP]) ammoidin; up to, imperatorin (ammid-in) and bergapten (heraclin, majudin, and 5-methoxy Psoralen [5-MOP]) and other coumarins of significance are marmesin (the structure of isolated compounds) isoimperatorin, heraclenin, and isopimpinellin constituents of interest are acetylated flavonoids [16-20]. The dried plant (500 g) of *A. majus L*. was sequentially extracted with hexane and methanol. In our initial biological study as shown in Table 1 the compounds **2**, **3 **showed high anti-inflammatory activity while the compound **4 **showed moderate activity. This effect could explain the medical use of *A. majus *in traditional medicine. The hexane extract was chromatographed to give β-sitosterol **1 **[21]. The methanol fraction was chromatographed on silica gel to give new coumarin **2 **and two coumarins **3**, **4**. Compound **2 **showed fluorescence under UV indicating it to be coumarin. The IR spectrum of the compound exhibited the presence of a carbonyl group at 1710 cm^-1 ^which was a further support toward the coumarin nucleus. MS suggested its molecular mass to be 206 in agreement with the formula C_11_H_10_O_4_, which shows fragments at *m/z *193 and 162, suggesting that fragmentation is occurring in the manner associated with coumarin nucleus. ^1^H NMR of the compound in CDCl_3 _showed that no band was typical of H-4 of a coumarin and singlet at δ 6.25 was assignable to H-3, indicating that methyl group was attached at position 4. Another doublet was observed at δ 6.62, which could be H-5 of a coumarin. There was a singlet at δ 6.43 and 6.82 for two protons which represented H-6 and H-8 of the nucleus. The ^13^C NMR spectrum showed resonance for all 11 carbon atoms in the molecule. The spectra revealed the presence of two methyl, three methane and six quaternary carbon atoms. The two downfield quaternary carbon signals at δ, 162.5 (C-3) and 143.7 (C-6) showed the presence of ketonic and one hydroxyl functionality in the molecule. The analytical results obtained from ^13^C NMR spectrum for this compound was tabulated in Table 2. Compound **3 **showed fluorescence under UV indicating it to be a coumarin. The IR spectrum of the compound exhibited the presence of a carbonyl group at 1700 cm^-1 ^which was a further support towards the coumarin nucleus. MS suggested its molecular mass to be 192 which agreement with formula C_10_H_8_O_4_. ^1^H NMR of the compound in CDCl_3 _showed a doublet at δ 6.72 which was typical of H-4 of a coumarin. Another doublet was observed at δ 5.35 which could be H-5 of a coumarin. There was a singlet at δ 6.25 and 6.80 for two protons which represented H-6 and H-8 of the nucleus. The analytical results obtained from ^13^C NMR spectrum for this compound were tabulated in Table 2. To the best of the authors' knowledge, the coumarin compound **3 **has not previously been isolated from this family. The ^1^H NMR data of furancoumarin system were closely similar to compound **4**, which included two doublets at δ 6.30 and 8.27 attributed to the pyran ring protons H-3 and H-4, two other doublets at δ 7.19 and 7.80 corresponding to the furan ring protons H-10 and H-9, and one olefinic proton at δ 7.20 (s) for H-8. The data proposed compound **4 **to be xanthotoxin [16-20].

**Table 1:** Anti-inflammatory data of the titled coumarin's compounds

Compounds	Thickness of rat paw (mm) after	
	
	3 h	% Inhibition after 3 h
**2**	0.43 ± 0.008^b^	37.81
**3**	0.41 ± 0.008^b^	36.80
**4**	0.53 ± 0.009^b^	28.17
Indomethacin	0.25 ± 0.1^b^	60.50
Control	0.72 ± 0.015	-

The results were significant.

Compared with control (carrogeenin only), ^a^*p *< 0.05

Compared with Indomethacin, ^b^*p *< 0.01

**Table 2:** ^1^H NMR and ^13^C NMR chemical shifts (*δ*/ppm) of (2) and (3) DMSO-*d_6 _*as the solvents (25°C)

Position	DMSO-*d_6_*			
	
	2		3	
	
	^**1**^**H**	^**13**^**C**	^**1**^**H**	^**13**^**C**
1	-	-	-	-
C2(C = O)	-	162.5	-	161.8
C3	6.29s	113.2	7.95d	117.05
C4	-	152.6	6.72d	147.3
C5	6.62d	109.3	5.35d	113.4
C6(OH)	6.43d	143.7	6.25d	143.6
C7	-	146.8	-	146.2
C8	6.82d	104.9	6.80d	104.2
C9	-	147.5	-	149.7
C10	-	112.5	-	111.3
C11(CH_3_)	2.45s	19.8	-	-
C12(OCH_3_)	3.84s	56.3	3.79s	56.1

### 2.2. Biological studies

#### 2.2.1. Anti-inflammatory activity

The pharmacological evaluation of the tested compounds (**2-4**) was carried out as per the protocol specified. The anti-inflammatory activity of the synthesized compounds was carried out using the carrageenan-induced rat paw edema method. The anti-inflammatory activity data for the compounds are given in Table 1. At the dose level of 0.01 mg/100 g, (**2**, **3**) exhibited appreciable inhibition of edema, especially **2**, which exhibited a 87% of edema inhibition of 37.81%, which was comparable to that of the standard drug indomethacin (60.50% at 0.01 mg/100 g dose) where the compound **4 **exhibited mild anti-inflammatory activity.

#### 2.2.2. Anti-viral activity

The compounds (**2-4**) found to have antiviral activity, [13] against vesicular stomatitis virus (VSV) in a concentration-dependent manner at complete non-toxic concentration range 10-100 μg/ml (Rf 10(5)), 10-100 μg/ml (Rf 10(4)), and 50-100 μg/ml (Rf 10(3)), respectively. All these compounds are found to have no reliable antiviral activity against herpes simplex virus (HSV).

## 3. Materials and methods

### 3.1. General

The ^1^H NMR and ^13^C NMR spectra were recorded at 270 and 68.5 MHz, respectively, with TMS as an internal standard using a 270-MHz JEOLJNM Ex-270/4000 NMR instrument. Optical rotations were determined on a JASCO P-1020 polarimeter using a 100-mm glass microcell. IR spectra (KBr) were recorded on a Perkin-Elmer 1650 FT-IR spectrometer. The UV spectra were recorded with a Perkin-Elmer Lambda 2UV/VIS spectrophotometer. The melting points were determined using a Digital Melting Point Apparatus (model IA 8103, Electro thermal Engineering Ltd, Soutthend-on-Sea, Essex, UK). MS were measured on a GSMS-QP-1000EX gas chromatograph-mass spectrometer SHIMADZU-Japan. For column chromatography, silica gel (Merk. 63-200 μm particle size) was used. TLC was carried out with Merk silica gel ^60^F_254 _Plates. UV light (245 and 366 mm) and spraying with vanillin-sulfuric acid reagent followed by heating (120 C) were used for detection.

### 3.2. Plant material

The aerial parts of the *A. Majus L*. were obtained from local market, Egypt, in March 2010. The plant material has been deposited at the Laboratory of Botany, Faculty of Science, and Zagazig University, Egypt.

### 3.3. Extraction and isolation

The air-dried plant (500 g) was powdered and extracted with hexane (1.6 l) at room temperature (25°C) for 30 min, and the hexane solution was evaporated *in vacuo *to give a residue (21 g). The methanol extract (32 g) was obtained by the same procedure. The hexane (20 g) was chromatographed over silica gel (200 g) using hexane with increasing amounts of ethyl acetate (5:1) to β-sitosterol (**1 **C_29_H_50_O). It is crystallized from methanol (20 mg; from Hexane-EtOAc 9:1, *R*_f _= 0.22 Light petroleum: EtOAc 2:1); mp 136°C (literature mp 136-137°C) [22]. It responded to Liebermann-Burchard Reaction. IRν_max _(KBr, cm^-1^) 3427; ^1^H NMR (δ, DMSO), 5.34 (1H, br, H-6), 3.51 (1H, m, H-3), 2.28-1.13 (29H, m, 11*CH_2_, 7*CH), 0.92 (6H, s, 2*CH_3_), 0.83 (3H, s, CH_3_), 0.80 (3H, s, CH_3_), 0.78 (3H, s, CH_3_), 0.68 (3H, s, CH_3_); GCMS: 414 (M+). This data confirmed compound **1 **to be β-sitosterol **1 **[21] using a direct comparison. The methanol extract (30 g) was chromatographed on a silica gel column using successively hexane-ethyl acetates eluent to give three coumarin compounds **(2-4)**.

### 3.4. 6-Hydroxy-7-methoxy-4 methyl coumarin (2 C_11_H_10_O_4_)

White, amorphous solid (53 mg; from CH_2_Cl_2_-EtOAc 8:2, *R*_f _= 0.19 Light petroleum: EtOAc 2:1); mp 204-206°C; [α]_D _+41.4 (CHCl_3_); UV 218; IR (KBr) γ_max _3620 (OH), 1710 (C = O) cm^-1^; ^1^H NMR and ^13^C NMR, see Table 2; *m/z *206 191(100), 160(17), 143(24); anal. calcd for C_11_H_10_O_4 _% C 64.06, % H 4.9, % O 31.3; found % C 64.03, % H 4.21, % O 31.1.

### 3.5. 6-Hydroxy-7-methoxy-coumarin (3 C_10_H_8_O_4_)

White, amorphous powder (61 mg; from CH_2_Cl_2_-EtOAc 3:1, *R*_f _= 0.16 Light petroleum: EtOAc 2:1); mp 183-185°C; [α]_D _+46.6 (CHCl_3_); UV 220; IR (KBr) γ_max _3640 (OH), 1700 (C = O); ^1^H NMR and ^13^C NMR, see Table 2; *m/z *192 177(17), 161(100) 144(25); anal. calcd for C_10_H_8_O_4 _% C 62.04, % H 4.21, % O 33.2; found % C 61.9, % H 4.43, % O 32.8.

### 3.6. Xanthotoxin (4 C_12_H_8_O_4_)

White, amorphous powder (26 mg; from CH_2_Cl_2_-EtOAc 1:1); mp 158-160°C; [α]_D _+46.6 (CHCl_3_). The data from IR (KBr), ^1^H NMR and ^13^C NMR proposed that compound **3 **is xanthotoxin [16-20]; anal. calcd for C_12_H_8_O_4 _% C 66.64, % H 3.71, % O 29.2; found % C 66.49, % H 3.43, % O 29.8.

### 3.7. Biological studies

#### 3.7.1. Anti-inflammatory activity

The anti-inflammatory activity was evaluated by hind paw oedema method [23]. Albino rats of weighing 100-150 g, of either three compounds **(2-4)**, using Indomethacin as a standard, were divided into five groups of six animals. The animals were maintained under normal environmental conditions. To each group, with the exception of the control group, the tested compounds (0.01 mg/100 g of body weight) were administered, injected. To one group, the standard drug Indomethacin (0.01 mg/100 g) was administered. After 1 h, carrageenan (0.1 ml, 1% w/v solution in sterile saline) was injected into the sub-plantar tissue of the left paw of all the animals. The right paw served as the reference non-inflamed paw for comparison. The initial paw volume was measured using a plethysmograph within 30 s of the injection. After 3 h, the final paw volume of each animal was measured. The percentage of reduction in the paw volume was calculated by subtracting the difference between the right and left hind paw volumes in the treated group from the difference in the control group and dividing it by the difference in the control group. The anti-inflammatory tivity of the tested compounds and the standard reference drug was determined using the formula, % anti-inflammatory activity = (1 - *V*_t_/*V*_c_) × 100, where *V*_t _represented the mean increase in paw volume of rats treated with test compounds and *V*_c _represented the mean increase in paw volume in the control group of rats.

#### 3.7.2. Anti-viral activity

In this study, the compounds **(2-4) **were evaluated for their anti-viral activity. These compounds were tested against two mammalian viruses, HSV-1 and VSV. The antiviral activity were determined by means of the end titration technique that depends on the ability of plant extract dilutions to inhibit the produced cytopathogenic effect and expressed as reduction factor (*R*_f_) of the viral titer.

## 4. Conclusion

*Ammi majus L*. being local medicinal plants with great abundance in west of Egypt are shown in rich in anti-viral and anti-inflammatory activities, including phytochemicals coumarin.{} These results give them the privilege to start intensive studies for isolation of these biologically active compounds for local drug-design programs. In addition, *A. majus L*. is considered as a good source of 6-hydroxy-7-methoxy coumarin **(3) **which was identified as the major coumarin. Also, this is the first study to report the occurrence of compound **(3)**.

## Competing interests

The authors declare that they have no competing interests.
